# A7 OPPORTUNISTIC PATHOGEN MODULATION OF GLUTEN-REACTIVE CD4+ T CELL ACTIVATION BY DQ2-EXPRESSING ORGANOID MONOLAYERS

**DOI:** 10.1093/jcag/gwac036.007

**Published:** 2023-03-07

**Authors:** S Rahmani, A Caminero, A Hann, H J Galipeau, R P Anderson, F Chirdo, T F Didar, E F Verdu

**Affiliations:** 1 Biomedical Engineering, Farncombe Family Digestive Health Research Institute; 2 Farncombe Family Digestive Health Research Institute , McMaster University, Hamilton, Canada; 3 Wesley Medical Research, The Wesley Hospital, Auchenflower, Australia; 4 Facultad de Ciencias Exactas, Universidad Nacional de La Plata, La Plata, Argentina; 5 Biomedical Engineering , McMaster University, Hamilton, Canada

## Abstract

**Background:**

Bacteria have recently emerged as additional modulators of inflammation in CeD. We have shown that the elastase-like producing opportunistic pathogen, *Pseudomonas (P) aeruginos*a, partially metabolizes gluten into peptides that translocate the mucosal barrier and retain their immunogenicity. We previously demonstrated that organoid monolayers derived from DR3-DQ2 mice carrying the CeD risk gene HLA-DQ2 express MHC class II (HLA-DQ2) and co-stimulatory molecules under induced inflammatory conditions, priming the monolayers for gluten antigen presentation. Here we investigate the activation of human (h)CD4^+^ T cell co-cultured with DQ2 monolayers stimulated with gluten pre-digested, or not, by bacterial elastase.

**Purpose:**

**To investigate whether organoid monolayers expressing DQ2 activate T cell differentially in the presence of gluten metabolized by elastase-like producing Pseudomonas aeruginosa.**

**Method:**

Organoid monolayers were derived from the duodenum and proximal jejunum of gluten-sensitized DR3-DQ2 mice, following the gluten sensitization protocol previously described^1^. Monolayers were then stimulated with IFN-γ for 24h to induce MHC-II and co-stimulatory molecules expression. Splenic T-cell expressing hCD4 from gluten-sensitized DR3-DQ2-hCD4 mice were then co-cultured with monolayers in the presence of deamidated pepsin-trypsin-digested (DAPT)-gluten or *Pseudomonas aeruginosa* PA14 (WT)-digested DAPT-gluten. As a control, DAPT-gluten was incubated with a *P. aeruginos*a *las*B mutant strain that lacks elastase-like activity (*las*B^△/△^). Co-cultures stimulated with DAPT-gluten alone or WT-media were used as additional controls.

**Result(s):**

Increased hCD4^+^ T-cell proliferation was observed in co-cultures stimulated with WT-digested gluten compared with *las*B^△/△^-digested gluten (*p<0.0001*), gluten alone (*p=0.0002*) or WT-media (*p<0.0001*). hCD4^+^ T cell co-cultured with organoid monolayers stimulated with WT-digested gluten, had an activated phenotype with increased expression of CD69, CD44 and CD25 versus those stimulated with gluten, *las*B^△/△^-digested gluten, or WT-media. Increased levels of pro-inflammatory and T helper type 1 (Th1)-associated cytokines were detected in the supernatant of the co-cultures stimulated with WT-digested gluten, including IL-2, IFN-γ, IL-6, TNF-α, IL-1α, IL-β, and IL-15.

**Conclusion(s):**

Using a novel HLA-DQ2-expressing organoid monolayer, we demonstrate elastase-like producing *P. aeruginosa,* enhanced activation and proliferation of hCD4^+^ T cell through gluten metabolism. This *in vitro* model constitutes a relevant tool for studying microbial triggers and drivers of intestinal epithelial dysfunction in CeD.

1. Galipeau, H. J. *et al.* 1. Galipeau, H. J. *et al.* Sensitization to Gliadin Induces Moderate Enteropathy and Insulitis in Nonobese Diabetic-DQ8 Mice. *J. Immunol.***187**, 4338–4346 (2011).

**Please acknowledge all funding agencies by checking the applicable boxes below:**

CIHR, Other

**Please indicate your source of funding;:**

Canadian Celiac Disease Association (CCA)

**Disclosure of Interest:**

None Declared

